# Liver metastasis from rectal neuroendocrine neoplasm detected 15 years after primary resection

**DOI:** 10.1186/s40792-022-01569-5

**Published:** 2022-12-02

**Authors:** Miho Akabane, Satoshi Okubo, Keiichi Kinowaki, Masaru Matsumura, Junichi Shindoh, Masaji Hashimoto

**Affiliations:** 1 Hepatobiliary-Pancreatic Surgery Division, Department of Gastroenterological Surgery, Toranomon Hospital, 2-2-2 Toranomon, Minatoku, Tokyo, 105-8470 Japan; 2 Department of Pathology, Toranomon Hospital, Tokyo, Japan; 3 Okinaka Memorial Institute for Medical Disease, Tokyo, Japan

**Keywords:** Neoplasm metastasis, Neuroendocrine tumors, Rectal neoplasms

## Abstract

**Background:**

Rectal neuroendocrine neoplasms can induce liver metastasis. However, few reports exist on the associated long-term recurrence rates. We report a case of liver metastasis identified 15 years after rectal neuroendocrine neoplasm resection.

**Case presentation:**

A 50-year-old woman was on semi-annual follow-up after undergoing mastectomy for breast cancer (pT1N0M0) and low anterior resection for grade 1 rectal neuroendocrine neoplasm (pT1b, ly1, v1). Fifteen years postoperatively, a 7-mm hyperechoic mass was identified at liver segment 6. Magnetic resonance imaging revealed a slight growth of the mass. Positron emission tomography/computed tomography revealed radiotracer accumulation in the lesion. Laparoscopic hepatectomy was performed. The histopathological diagnosis was grade 2 neuroendocrine neoplasm. The pathological findings and clinical course indicated the tumor originated in the rectum.

**Conclusions:**

Our findings highlight the need to reassess the optimal postoperative follow-up period for patients with rectal neuroendocrine neoplasm.

## Background

Liver metastasis from rectal neuroendocrine neoplasms (NENs) can occur even after curative resection. The recommended postoperative follow-up duration depends on the presence of risk factors for recurrence [1]. Herein, we describe a case of detection of liver metastasis from rectal NEN 15 years after radical resection with lymphadenectomy; the metastasis could have been missed if the recommended follow-up protocol was followed. This case is noteworthy as it can inspire discussion on the optimal postoperative follow-up duration.

## Case presentation

**Fig. 1:**
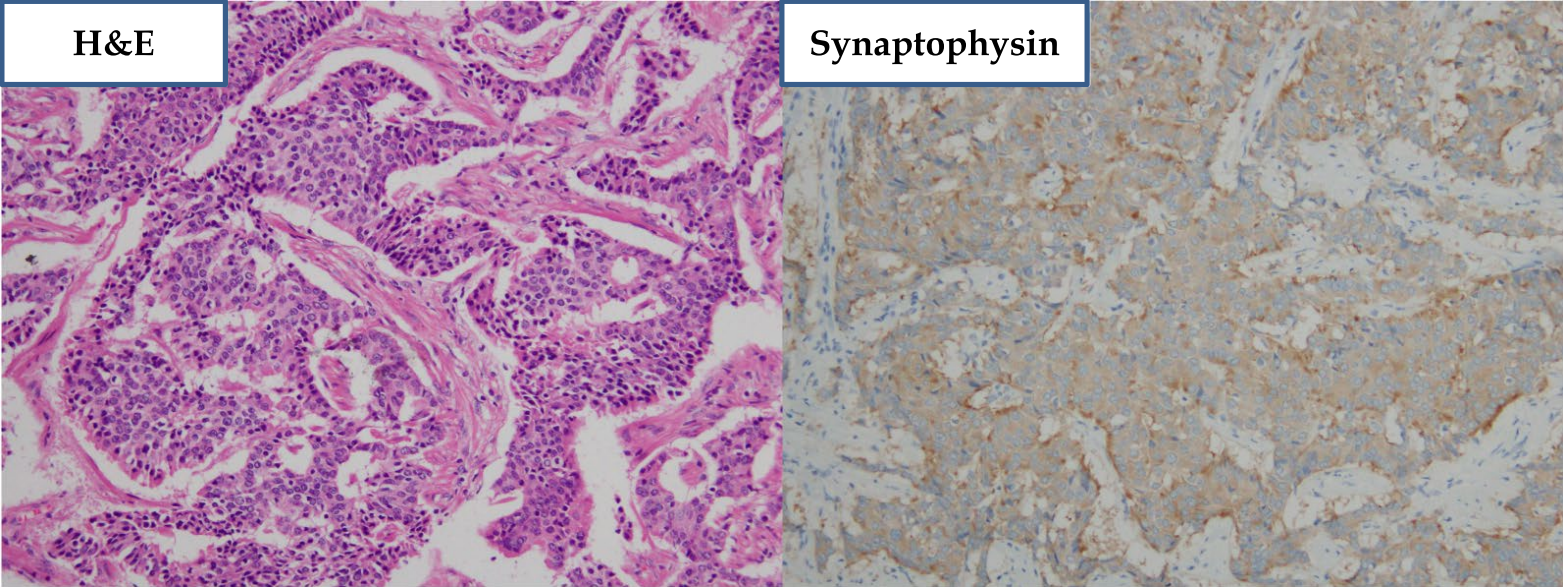
Surgical specimen of rectal neuroendocrine neoplasm. The tumor cells show anisokaryosis and a trabecular formation (hematoxylin and eosin staining, ×200), and are positive for synaptophysin (×200)

**Fig. 2:**
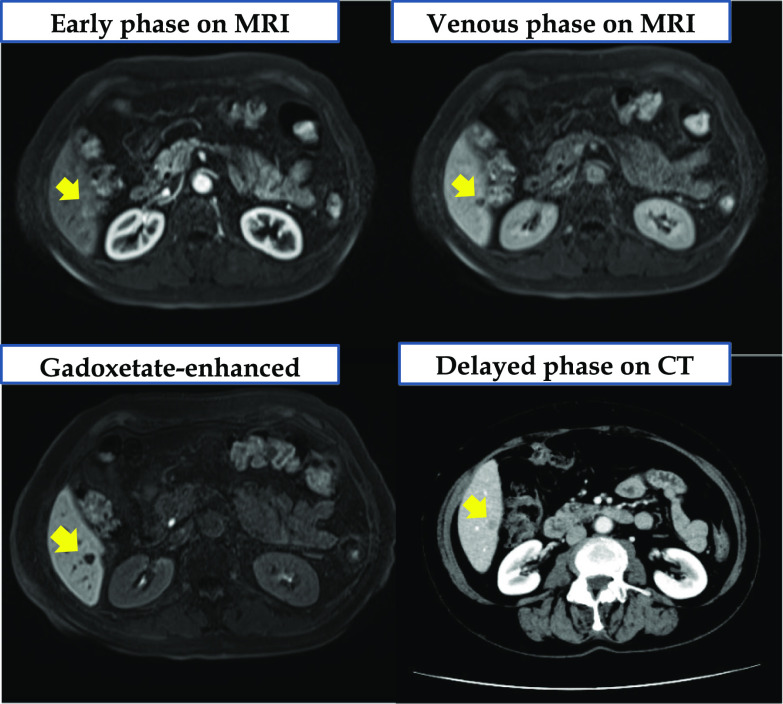
Magnetic resonance image and computed tomography obtained at the 15-year follow-up. On magnetic resonance image, the mass is enhanced in the early phase (arrow) and washed out in the venous phase (arrow). The uptake of gadoxetate sodium is decreased (arrow). On computed tomography, a hypovascular nodule is seen in the delayed phase (arrow)

A 50-year-old woman underwent left total mastectomy for breast cancer (pT1N0M0 by AJCC-8th) and low anterior resection for a localized NEN. A 10-mm mass was observed at the lower rectum. Submucosal and lymphovascular invasion (ly1, v1) with negative surgical margins were noted. The mitotic count was < 1 per 10 high-power fields. Immunohistochemical staining revealed the cells were positive for synaptophysin, chromogranin A, and CD56 (Fig. 1). The Ki-67 labeling index was < 2%. Therefore, rectal NEN (grade 1) was diagnosed. Postoperatively, she was on semi-annual follow-up for imaging studies. At the 15-year follow-up, a 7-mm hyperechoic hepatic mass was identified. Magnetic resonance imaging (MRI) depicted a 7-mm nodule at segment 6 with high signal intensity on T2-weighted and diffusion-weighted images and decreased gadoxetate sodium uptake. It was enhanced in the early phase and washed out in the venous phase. Three months later, MRI revealed an increase in size to 9 mm (Fig. 2). Contrast-enhanced computed tomography (CT) revealed a hypovascular nodule (Fig. 2). Positron emission tomography/CT revealed radiotracer accumulation (maximum standardized uptake value: 3.74) at the mass. There was 7% indocyanine green retention at 15 min. Laboratory findings indicated normal renal and liver function. She had no hepatitis. All the electrolytes and serum tumor markers were within normal ranges. The Child–Pugh class was A, and she never drank alcohol. Based on these findings, metastasis from breast cancer was considered the most likely diagnosis. The differential diagnoses included metastasis from rectal NEN and hepatocellular carcinoma.

Therefore, surgical resection was planned. Ascites, dissemination, and distant metastasis were not observed. Intraoperative ultrasonography revealed a single mass located at segment 6. No other hepatic lesion was identified. Laparoscopic partial hepatectomy was performed. The operation took 98 min. She was discharged on postoperative day 5 after uneventful postoperative course.

**Fig. 3:**
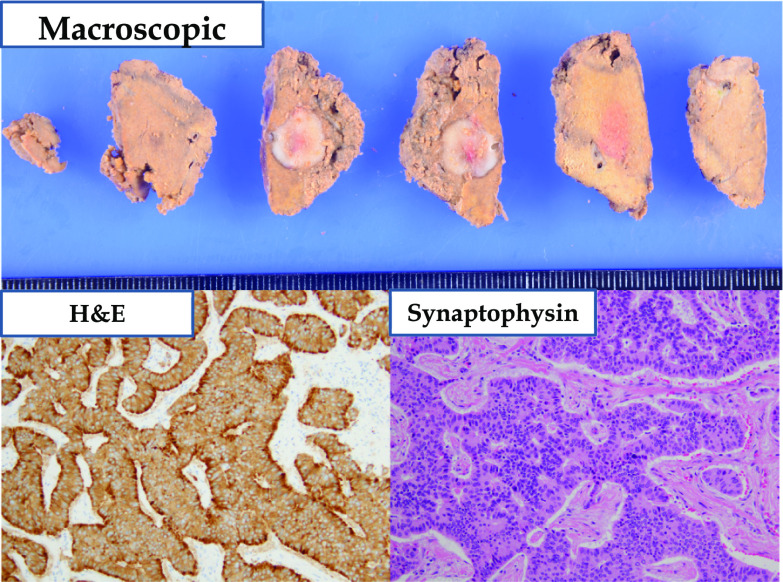
Surgical specimen of hepatic mass. The specimen is an 11.0 × 10.0 × 10.0 mm white mass. Atypical cells with round nuclei are seen arranged in a rosette-like pattern (hematoxylin and eosin staining, ×200). The cells are positive for synaptophysin (×200)

Gross examination revealed an 11.0 × 10.0 × 10.0-mm mass. Histological examination revealed atypical cells with round nuclei arranged in a rosette-like pattern (Fig. 3). The mitotic count was 3–4 per 10 high-power fields. The cells were positive for chromogranin A, synaptophysin, and CD56. The Ki-67 labeling index was 4.06%. Based on the pathological findings and clinical course, metastasis from rectal NEN (grade 2) was diagnosed. Postoperatively, regular follow-up for imaging studies is being performed. At the 6-month follow-up, the patient was recurrence-free.

## Discussion

NENs originate from the diffuse system of neuroendocrine cells. The most common primary site of NENs is the gastrointestinal tract (67.5%) [2]. The small intestine (25.3%) and rectum (27.4%) are the most common gastrointestinal origin sites [2].

This case can inspire discussion on the origin of hepatic NENs: primary or metastatic. Primary hepatic NENs are extremely rare (0.4%) [2], and their diagnosis requires exclusion of other primary lesions. Additionally, the liver is the most common metastatic site. Approximately 75% of all NENs can metastasize to the liver [3]. Furthermore, primary hepatic NENs are usually hypervascular [4]. However, CT revealed our patient’s tumor was hypovascular (Fig. 2). Moreover, pathological examination revealed similar immunohistological patterns, nuclear sizes, and mitotic counts between the rectal and hepatic specimens. Therefore, metastatic hepatic NEN was diagnosed.

We searched the English literature for reports of liver metastasis identified after a recurrence-free period of ≥ 5 years after rectal NEN resection [5–7] (Table 1). The European Neuroendocrine Tumor Society (ENETS) guidelines recommend endoscopic or local resection for tumors < 10 mm in size. For 10- to 20-mm-sized tumors that are confined to the submucosa, local resection is preferred, and anterior resection with lymphadenectomy is only preferred if the tumor invades the muscularis propria. For grade 3 tumors that are 10–20 mm or tumors larger than 20 mm, anterior resection with lymphadenectomy is recommended if there is no distant metastasis [1]. In the present case, low anterior resection with prophylactic lymphadenectomy was performed, because the tumor size had been expected to be 10 mm or more based on the preoperative endoscopic findings and the possibility of the existence of lymph node metastasis could not be ruled out. Recurrence occurred despite appropriate treatment according to ENETS recommendation in all previous cases except those reported by Hane et al. [5]. Recurrence and survival in patients with rectal NEN depends on the tumor size, tumor depth, lymphovascular invasion, and mitotic rate [8]. Based on the tumor size (≥ 10 mm) and lymphovascular invasion status, our patient can be considered at intermediate risk, which suggests a possibility of distant metastasis in the future. The National Comprehensive Cancer Network recommends a proctoscopic examination be conducted at 6 and 12 months postoperatively in patients with 10–20 mm-sized rectal NENs [9]; in this case, however, this follow-up period was insufficient. Furthermore, according to ENETS guidelines, patients with completely resected rectal NENs that are > 10 mm in size should undergo surveillance rectoscopy 1 year, 3 years, and then every 5 years postoperatively [1]; however, following this protocol could have resulted in us missing the liver metastasis. It is noteworthy that despite performing annual follow-up imaging studies, distant metastasis was identified for the first time after an extremely long observation period of 15 years. We usually follow up patients who have undergone surgery for rectal NEN according to the National Comprehensive Cancer Network clinical guideline [9]. As per the postoperative follow-up for colorectal cancer, the follow-up term for rectal NET in our facility is generally 5 years, and abdominal ultrasonography and plain CT are alternately performed semi-annually to search for possible distant metastasis. After 5 years, additional annual follow-up will be considered in consultation with the patients. Kwann et al. reported that the median follow-up for endoscopically treated patients was 2.0 years (range, 0–16 years; mean [SD], 3.6 [4.1] years) [7]. Although Shigematsu et al. reported the longest interval to recurrence among the previous cases, they seem to have conducted annual follow-up only after incidental identification of liver metastasis during a medical checkup 2 years prior [6], which suggests the liver metastasis might have occurred long before it was identified. Sugimoto et al. reported the Ki-67 index (> 3%) and lymphovascular permeation were reliable predictive markers for rectal NEN metastasis [10]. Our patient had a relatively low Ki-67 score. Thus, we can speculate lymphovascular invasion played an important role in provoking distant metastasis in this case. However, cases of recurrence without lymphovascular invasion have been reported [5, 6]. Therefore, existing risk factors alone may not be sufficient to identify patients at risk of distant metastasis. A long observation period may be needed to avoid missing distant metastasis. Considering these reports, patients with risk factors for distant metastasis might require annual imaging studies for at least 15 years postoperatively (Table 1).

**Table 1:** Existing reports describing liver metastasis after a recurrence-free period of at least 5 years after resection of rectal NEN

	Recurrence-free period (years)	Surgical margin	Rectal tumor size (mm)	Ki-67 (%)	Tumor depth	Treatment for rectal NEN	Lymphovascular invasion	Number of liver metastasis	Maximum size of liver metastasis (mm)	Treatment for liver metastasis	Outcome after treatment of liver metastasis
Shigematsu et al. [6]	30	Negative	10	5.0	SM	Transanal posterior proctotomy	Absent	Multiple	50	Hepatectomy	Recurrence at the 1-year follow-up
Kwaan et al. [7]	13	Negative	6	N/A	SM	Transanal resection	N/A	Multiple	N/A	N/A	N/A
Hane et al. [5]	13	Negative	12	1.7	SM	Endoscopic resection	Present	Multiple	47	Everolimus	Stable disease at the 39-month follow-up
Hane et al. [5]	10	Negative	13	< 1.0	SM	Endoscopic resection	Absent	Multiple	N/A	Octreotide/everolimus/streptozocin	Died of liver metastasis at the 1-year follow-up
Hane et al. [5]	9	Negative	10	5	SM	Endoscopic resection	Absent	Multiple	15	Radiofrequency ablation + octreotide	Recurrence at the 1-year follow-up
Kwaan et al. [7]	5	Positive	7	N/A	SM	Endoscopic resection	N/A	Multiple	N/A	N/A	N/A
Our case	15	Negative	10	< 2.0	SM	Low anterior resection + lymphadenectomy	Present	Single	7	Hepatectomy	Recurrence-free at the 6-month follow-up

*NEN* neuroendocrine neoplasm, *SM* submucosa, *MP* muscularis propria, *N/A* not applicable

## Conclusions

Our case has two remarkable features: (1) the extremely long observation period after surgical resection and (2) the implication that postoperative follow-up should be performed for a considerably long period in patients with risk factors for recurrence.

## Data Availability

All data generated or analyzed during this study are included in the published article.

## Abbreviations

NENs: Neuroendocrine neoplasms
MRI: Magnetic resonance imaging
CT: Computed tomography
ENETS: European Neuroendocrine Tumor Society
